# Correction: Kao et al. Pulmonary Fat Embolism Following Liposuction and Fat Grafting: A Review of Published Cases. *Healthcare* 2023, *11*, 1391

**DOI:** 10.3390/healthcare12131328

**Published:** 2024-07-03

**Authors:** Yu-Ming Kao, Kuo-Tai Chen, Kuo-Chang Lee, Chien-Chin Hsu, Yeh-Cheng Chien

**Affiliations:** 1 Division of General Surgery, Department of Surgery, Chi-Mei Medical Center, Tainan 71004, Taiwan; 2 Emergency Department, Chi-Mei Medical Center, Tainan 71004, Taiwan; 3 Emergency Department, Chi-Mei Medical Center Chiali Branch, Tainan 71004, Taiwan; 4 Department of Biotechnology, Southern Tainan University of Technology, Tainan 71005, Taiwan

In the original publication [1], there were mistakes in Figure 1 and Figure 2 and Table 1, Table 2 and Table 3 as published due to the following reasons.

First, we removed 2019, Foula A [42] due to a duplicated publication and 2010, Coro-nado-Malagón M [27] because this case did not undergo liposuction and only underwent the injection of a soft tissue filler. With this correction, the order of some references has been adjusted accordingly.

Second, 12 expired cases were incorrectly marked as alive in Table 1. 

Third, six cases actually did not undergo fat grafting, encompassing Laub Jr and Laub, 1990 [19], Erba et al., 2011 [28], Saon et al., 2019 [44], Kadar et al., 2021 [13], Foula et al., 2022 [47], and Pham 2022 [48].

Because of these data corrections, the results of this manuscript should be revised.

**Error in Figure/Table**

The corrected Figure 1 and Figure 2 and Table 1, Table 2 and Table 3 appear below. 

**Figure 1:**
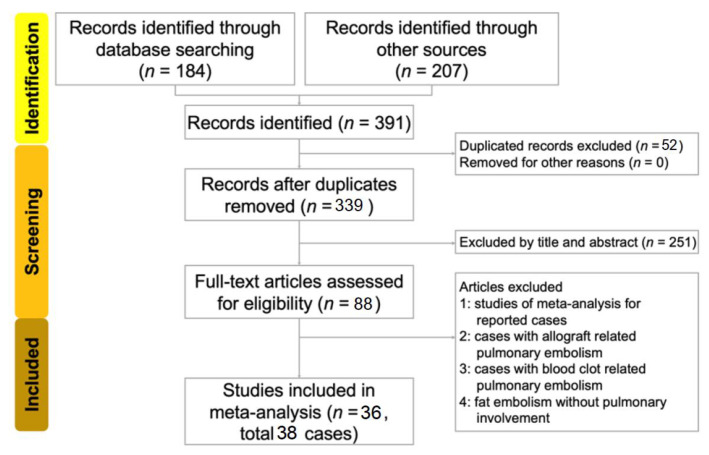
Flow diagram for the search and identification of included studies and patients.

**Figure 2:**
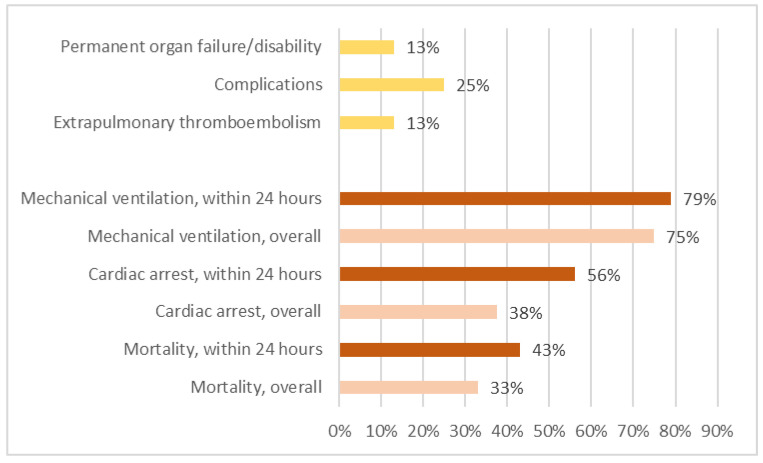
Mortality, cardiac arrest events, and mechanical ventilation rates for all patients and for patients with symptoms onset within 24 h after surgery. We excluded the deceased and calculated rates for extrapulmonary thromboembolism, complications, and permanent organ failure/disability.

**Table 1:** Reviewed studies and patients’ characteristics.

Year, Author	Country	Sex	Age	Surgery	Cardiac Arrest	Mechanical Ventilation	Mortality
1983, Hunter GR [15]	US	F	37	Liposuction	No	Unknown	Alive
1986, Christman KD [16]	US	F	56	Liposuction	Yes	Yes	Dead
1988, Ross RM [17]	US	F	44	Liposuction	No	Yes	Alive
1990, Boezaart AP [18]	South Africa	F	39	Liposuction	No	Yes	Alive
1990, Laub Jr DR [19]	US	F	51	Liposuction	No	No	Alive
1997, Currie I [20]	Canada	F	69	Liposuction, fat grafting	Yes	Yes	Dead
1998, Fourme T [21]	France	F	29	Liposuction	No	No	Alive
1999, Folador JC [22]	Brazil	F	40	Liposuction	No	No	Alive
1999, Scroggins C [23]	US	F	54	Liposuction	No	Yes	Alive
2002, Platt MS-1 [14]	US	F	82	Liposuction	Yes	Yes	Dead
2002, Platt MS-2 [14]	US	M	50	Liposuction	Yes	Yes	Dead
2006, Rothmann C [24]	France	F	24	Liposuction	No	Yes	Alive
2007, Wessman DE [25]	US	M	31	Liposuction	No	No	Alive
2008, Costa AN [26]	Brazil	M	53	Liposuction, fat grafting	No	Yes	Alive
2011, Erba P [27]	Switzerland	F	46	Liposuction	No	Yes	Alive
2011, Gleeson CM [28]	UK	F	37	Liposuction, fat grafting	Yes	Yes	Dead
2012, Shiffman MA [29]	US	F	40	Liposuction, fat grafting	Yes	Yes	Dead
2013, Zeidman M [30]	US	F	24	Liposuction	No	Yes	Alive
2014, Cohen L [31]	US	F	58	Liposuction	Unknown	Unknown	Unknown
2014, Hostiuc S [32]	Romania	F	56	Liposuction	Yes	Yes	Dead
2015, Astarita DC [33]	US	F	42	Liposuction, fat grafting	Yes	Yes	Dead
2015, Byeon SW [34]	Korea	M	21	Liposuction	No	Yes	Alive
2015, Cárdenas-Camarena L [35]	Colombia	F	37	Liposuction, fat grafting	Yes	Yes	Dead
2015, Fu X [36]	China	F	30	Liposuction, fat grafting	No	No	Alive
2015, Vidua RK [37]	India	F	39	Liposuction	Yes	No	Dead
2016, Souza RL [12]	Brazil	F	42	Liposuction, fat grafting	No	Yes	Dead
2017, Ali A [38]	UK	F	45	Liposuction	No	Yes	Alive
2017, Sasaki Y [39]	Japan	F	29	Liposuction	No	Yes	Alive
2017, Zilg B [40]	Sweden	M	31	Liposuction, fat grafting	Yes	Yes	Dead
2019, Peña W [41]	Mexico	F	41	Liposuction, fat grafting	Yes	Yes	Alive
2019, Saon MD [42]	US	F	52	Liposuction	No	No	Alive
2020, Recinos S [43]	Guatemala	M	37	Liposuction, fat grafting	Yes	Yes	Alive
2021, Kadar A [13]	US	F	26	Liposuction	No	Yes	Alive
2022, Fonseca EKUN [44]	Brazil	F	32	Liposuction	Unknown	Unknown	Unknown
2022, Foula AS [45]	Egypt	F	29	Liposuction	Yes	Yes	Alive
2022, Pham MQ [46]	Vietnam	F	37	Liposuction	No	No	Alive
2022, Wolfe EM-1 [47]	US	F	28	Liposuction, fat grafting	No	Yes	Alive
2022, Wolfe EM-2 [47]	US	F	26	Liposuction, fat grafting	No	Yes	Alive

**Table 2:** Demographic data, types of surgery, and body parts.

Demographic Data	Percentage	Body Parts	Percentage
Age (years)	39.0 (30.3–49.0) *	Abdomen/flank	21 (55%)
Sex (female)	32 (84%)	Lower limbs	14 (37%)
Comorbidity	7 (18%)	Buttocks	12 (32%)
Surgery		Breast/chest	9 (24%)
Liposuction	38 (100%)	Upper limbs	3 (8%)
Fat grafting	13 (34%)	Head/neck	2 (5%)
Others #	3 (8%)	Penis	2 (5%)

* Median (interquartile range). # Others: repairs of diastasis rectus in two patients, and hysterectomy and oophorectomy in one patient.

**Table 3:** Symptoms, laboratory tests, and diagnostic measurements of the included patients. In some studies, patient characteristics were not described; therefore, the sum for each item may not be 40.

Symptoms	Percentage	Diagnostic Measurements	Percentage
Dyspnea	21 (55%)	Examinations	
Hypotension	16 (42%)	CT scan	18/18 (100%)
Tachycardia	14 (37%)	CXR	16/19 (84%)
Hypoxia	12 (32%)	Echocardiogram	8/12 (67%)
Altered mental state	9 (24%)	Bronchoalveolar lavage	4/4 (100%)
Cardiac arrest	8 (21%)	Autopsy	10/10 (100%)
Fever	7 (18%)	Pulmonary angiogram	3/3 (100%)
Skin rash/petechiae	5 (13%)	Laboratory tests	
Chest pain	4 (11%)	PaO_2_/FiO_2_ ≤ 200 mmHg	18/18 (100%)
Cyanosis	4 (11%)	White cell count > 1000, < 4000/μL	9/11 (82%)
Cough	3 (8%)	Hemoglobin < 12 g/dL	8/14 (57%)
Hemoptysis	3 (8%)	Platelet < 150,000/μL	5/11 (45%)
Syncope	2 (5%)	Creatine > 1.2 g/L	2/5 (40%)
Bradycardia	2 (5%)	T bilirubin > 1.2 g/L	2/3 (67%)
Neurologic deficit	2 (5%)	D-dimer > 500 mg/L	4/4 (100%)

**Text Correction**

There was an error in the original publication because of the reasons mentioned previously.

A correction has been made to the Abstract and the Results, page 1.

A total of 38 patients from 20 countries were included. Chest computed tomography (CT) yielded 100% accuracy in the diagnosis of PFE. All of the deceased died within 5 days after surgery, and in 76% of patients, onset of symptoms occurred within 24 h after surgery. The proportions of patients who required mechanical ventilation, had a cardiac arrest event, or died among all patients and among those whose onset of symptoms occurred within 24 h after surgery were 75%, 38%, and 33% versus 79%, 56%, and 43%, respectively.

A correction has been made to the Results, pages 3–7.

*3.1.* *Characteristics of Enrolled Studies*

We identified 184 studies from the database search and 207 studies from reference lists, resulting in a total of 391 studies. A total of 52 studies were excluded as duplicates. The titles and abstracts of the remaining 340 studies were reviewed. After excluding 251 studies, the authors meticulously reviewed the remaining 88 studies. Of these, 52 were excluded. Among these 52 excluded studies, 5 were meta-analyses, 8 involved allograft-related pulmonary embolism, 4 involved blood-clot-related pulmonary embolism, and 35 involved fat embolisms without pulmonary involvement. Finally, 36 studies (33 in English, 1 in Japanese, 1 in French, and 1 in Portuguese) involving 39 patients met our search criteria. We excluded the case of one patient in a collected study because it contained no evidence of PFE [14]. A total of 38 patients were included in our review. A flow diagram of the search and identification strategy is presented in Figure 1. Patient characteristics are presented in Table 1.

The study included a total of 38 patients, of which 84% were women and 82% had no comorbidities. The age of patients ranged from 21 to 82 years. Liposuction was performed in all studies, and fat grafting was performed in 34% of studies. It is impossible to distinguish the risk of PFE between patients with liposuction alone and those who also received fat grafting. In most cases, the dimensions of the cannula used during surgery were not recorded. The volume of solution aspirated during liposuction varied widely, ranging from 35 to 10,000 mL, and the most frequently treated body parts were the abdomen/flank, lower limbs, buttocks, and breast/chest (as shown in Table 2).

In total, 16 patients were in the United States, 4 were in Brazil, 2 were in France, 2 were in the United Kingdom, and 1 patient each was reported in Canada, China, Colombia, Egypt, Guatemala, India, Japan, Korea, Mexico, Romania, Saudi Arabia, South Africa, Sweden, Switzerland, Taiwan, and Vietnam.

*3.2.* *Symptoms, Laboratory Tests, and Diagnostic Measurements of Enrolled Patients*

Common symptoms included dyspnea, hypotension, tachycardia, hypoxia, and altered mental state. A total of 21% of patients presented initially with cardiac arrest events. The time to symptom onset ranged from 0 to 13 days after surgery. Most patients (76%) had onset of symptoms within 24 h after surgery, and the time to symptom onset was not reported for one patient.

In terms of laboratory tests, all patients had decreased PaO_2_/FiO_2_ ratios (PaO_2_/FiO_2_ ≤ 200). Additionally, leukocytosis, anemia, thrombocytopenia, and elevation of D-dimer were frequently observed abnormalities. 

Autopsy, chest CT, and bronchoalveolar lavage yielded high accuracy in the diagnosis of PFE. Microscopic examination during autopsy typically shows multiple adipose tissue emboli in pulmonary arteries [40]. Chest CT scan commonly reveals diffuse mixed ground-glass and consolidative opacities involving both lungs. Bronchoalveolar lavage fluid typically contains blood-tinged secretions in the airways, and microscopic examination of the specimens typically reveals the presence of lipid-laden macrophages [13]. Before 1997, pulmonary angiograms revealed multiple irregular peripheral defects in the pulmonary arterial tree with subsegmental occlusions indicating micro emboli in three patients [16,19,20]. The majority of reported plain chest X-ray (CXR) findings revealed opacifications of bilateral lung fields, similar to the presentation of pulmonary edema or adult respiratory distress syndrome. However, 16% of the reported CXR findings were negative. In the acute stage, 67% of echocardiograms revealed abnormalities, including global hypokinesia, dilated right ventricle, or signs of pulmonary hypertension [28,34]. Symptoms, laboratory abnormalities, and various diagnostic measurements are listed in Table 3.

*3.5.* *Outcome*

The outcome of a patient was not described in two studies [31,44]. Therefore, 36 patients were included for outcome analysis. In total, 12 patients died, all within 5 days after surgery. Among the 37 patients, 76% required mechanical ventilation, 38% had cardiac arrest events, and 33% died. Among patients whose symptoms onset within 24 h after surgery, 75% required mechanical ventilation, 38% had cardiac arrest events, and 33% died (Figure 2).

Extrapulmonary thromboembolisms were commonly discovered during autopsy and imaging studies. Among the extrapulmonary thromboembolisms that were discovered, four were cerebral embolisms, two were retinal embolisms, two were lower limb venous thromboembolisms, one was a renal embolism, and one was a spleen embolism. However, among the 24 patients who survived, only 3 (13%) had extrapulmonary thromboembolisms. Complications, including three wound infections, one lung infection requiring lobectomy, one hypoxic encephalopathy, and one acute renal failure, were present in six patients (25%). Extrapulmonary thromboembolisms and complications were present in three patients (13%) with permanent organ failure and disability, including one case of blindness due to a retinal fat embolism, one disability related to cerebral infarction, and one renal failure requiring long-term hemodialysis. Figure 2 shows the incidences of mortality, cardiac arrest events, mechanical ventilation, extrapulmonary thromboembolism, complications, and permanent organ failure/disability of varied patient groups.

The authors state that the scientific conclusions are unaffected. This correction was approved by the Academic Editor. The original publication has also been updated.
